# Genetic and Genomic Web Resources for Research on Alcohol Use and Abuse

**Published:** 2012

**Authors:** Robert W. Williams

**Affiliations:** **Robert W. Williams, Ph.D.,***is a professor in the Center for Integrative and Translational Genomics, The University of Tennessee Health Science Center, Memphis, Tennessee.*

There are two major ways of publishing scientific data and results: (1) the standard peer-reviewed paper, which dates back to volume 1 of the Philosophical Transactions of the Royal Society in 1665; and (2) online distribution of data, resources, and software using the Internet that dates back a mere 21 years to the first Web site at the European Organization for Nuclear Research (CERN) established by Tim Berners-Lee.

Today online resources for sharing scientific work abound. The National Library of Medicine’s repository, PubMed, captures more than 21 million citations for biomedical literature. NIAAA can lay claim to the first URL listed in PubMed: The Portable Dictionary of the Mouse Genome (Williams 1994). This site—now called GeneNetwork.org—has been supported by NIAAA for more than a decade as part of the Integrative Neuroscience of Initiative on Alcoholism (INIA).

There are hundreds of sophisticated Web services and resources that can be exploited by students and researchers interested in alcoholism and other substance use disorders. These resources can be used like publications, but a better way to think about them is as a second “dry” laboratory in which it is possible to carry out experiments and to either generate or test ideas by reusing data that often have been rescued from the classic literature.

Below is a short list of both well-known and more esoteric resources, many of which have been supported by NIAAA, that can be used as a complement to the set of reviews in this special issue. There are two major categories of sites in this list: (1) those that provide deep data along with software that can be used to perform analysis, (2) those that can provide physical resources such as samples, clones, and powerful experimental murine models. The first category is easy to browse directly from the links below; whereas the second category is geared more to students and scientists in need of a jump-start to understand the function of specific genes.

## Category 1: Web Resources for Online Analysis of the Genetics of Alcoholism and More

### GeneNetwork (www.genenetwork.org):

This is a comprehensive resource for learning about genetics, but users may need to read the help files, FAQs, or one of the references (Chesler et al., 2003; Grisham et al., 2010, www.lifescied.org/content/9/2/98.full.pdf). GeneNetwork is one of an interlinked trio of sites built up by NIAAA (GeneWeaver and WebGestalt are the other two) to house extensive data for human, monkey, rat, mouse, and fruit fly. It includes hundreds of data sets on responses to alcohol, particularly in a family of mice called the BXDs. Data are linked with powerful gene analysis and mapping tools. Think of it as a free suite of genetics and statistics programs that happen to be loaded with genetic and genomic data sets, along with complimentary data on biological responses to alcohol and many other drugs (Philip et al., 2010). (For more information, see figure 1A and B)

### GeneWeaver (www.geneweaver.org):

**Figure 2:**
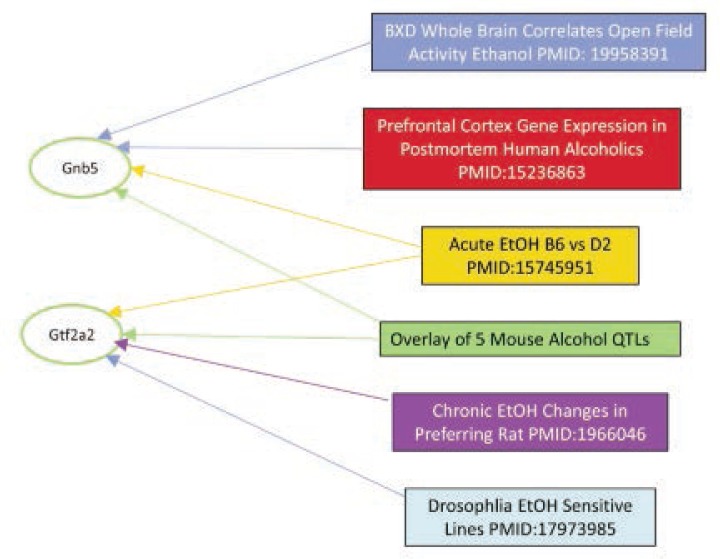
GeneWeaver output graph illustrating this program’s ability to find the connections between six data sets listed to the right in boxes with two interesting candidate genes—GNB5 and GTF2A2 (left side in ovals). These two candidate genes are both located in the region highlighted in the QTL map of Figure 1B.

This is another NIAAA-funded project that offers a powerful tool for the integrative analysis of collections of lists of genes and their functional relationships (Baker et al., 2012). This resource-and-analysis tool provides a way of making sense of a large group of related genomic studies. Excellent user interface and tutorials make this a starting point for those with large gene expression data sets. It also is a straightforward of performing analyses of many curated gene sets in the GeneWeaver database (see figure 2).

### WebGestalt (http://bioinfo.vanderbilt.edu/webgestalt):

Like GeneWeaver this is a sophisticated tool for the analysis of sets of genes. It includes species as diverse as yeast, worms, and humans (Duncan et al., 2010). WebGestalt often is used to perform pathway analysis and gene ontology analysis—a computationally demanding categorization of genes based on their known functions.

### Allen Brain Atlas (www.brain-map.org):

This site is a noteworthy philanthropic contribution from Paul Allen to brain research. The site started with a focus on gene expression patterns in the brain of the mouse; however, within the last year, it has expanded rapidly and now also covers gene expression in humans and non-human primates. Scientists interested in brain research should visit this site at least once to see the full power of Web services and Web science—it puts a massive research lab at your fingertips.

### GeneMania (www.genemania.org) and Gemma (www.chibi.ubc.ca/Gemma):

These are two Web sites used for the systematic analysis of molecular networks. Both of these Canadian sites have powerful and user-friendly interfaces.

### Mouse Phenome Database (http://phenome.jax.org):

This database offers medical records for mice, including mice that will voluntarily drink to intoxication. The site includes hundreds of valuable phenotype data for generations of mice (Maddatu et al., 2012). It includes links to matched genetic data.

### PhenoGen Informatics (http://phenogen.ucdenver.edu):

This site provides deep and total access to many massive microarray data sets, many with a focus on the genetics and genomics of alcoholism (Bhave et al., 2007). The site can be used to analyze scientists’ own data sets, particularly microarray data.

### Collaborative Study on the Genetics of Alcoholism (http://zork.wustl.edu/niaaa/):

COGA might be considered the “mother lode” of studies on the genetics of alcoholism, with more than 300 families and 3,000 individuals. This remains an active research program. Because of patient confidentiality it is not possible to directly access key data, as is the case for mouse and rat resources. Still this site provides a comprehensive overview of the data that have been generated and links to virtually all of the associated research papers.

### Portland Alcohol Research Center (http://www.ohsu.edu/parc/by_phen.shtml):

This site contains a useful and extensive list of gene regions known to modulate response to alcohol in mouse models. A number of these loci have been “converted” into single causal gene variants and many more should now be aligned to corresponding regions in the mouse genome. (See the next resource—the ERGR—for online comparison of the human genome to those of other species.)

### Ethanol-Related Gene Resource (http://bioinfo.mc.vanderbilt.edu/ERGR/):

This is an excellent site for reviewing what is known about the genetics of alcoholism in many different types of organism—offering a comparative approach (Guo et al., 2009).

## Category 2: Web Resources for Tissues, Clones, and Mouse Models

### MATRR (https://gleek.ecs.baylor.edu/):

The Monkey Alcohol Tissue Research Resource is a new NIAAA-supported site intended for experts in the field who need to understand the causes and impact of alcoholism. Non-human primates have proved to be strikingly faithful models of many aspects of alcoholism in humans, and they provide far better experimental control.

### Gene Knockout Project (KOMP and EUCOMM, at www.knockoutmouse.org):

This is an international initiative that is making major mutations (knock-outs) in every one of about 19,000 genes in the mammalian (murine) genome. NIAAA contributes to this effort, with a special focus on those genes known to be involved in brain function and suspected to modulate risk of alcoholism.

### Cre-driver Network (www.credrivermice.org):

This is a companion to the knockout project (above). It provides information on how to obtain lines of mice that can be used to turn genes off in specific types of cells at different points in life.

## Figures and Tables

**Figure 1A:**
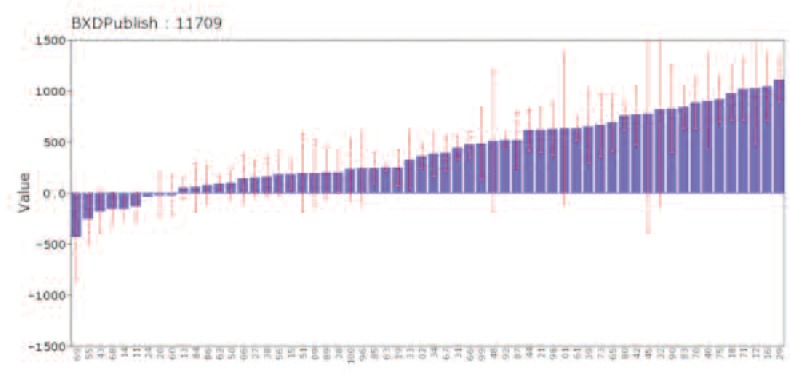
Effects of alcohol taken from mice. This plot is taken directly from GeneNetwork and the work of Philip and colleagues (2010). It illustrates the high variability in the response to alcohol among 60 strains of mice (adult females, GeneNetwork Trait Mouse BXD Published Phenotype 11709). Differences in the height of the blue bars highlight the substantial genetic variation in activity levels (sedation to the left, activation to the right) among strains. The red error bars highlight the important role of non-genetic factors. Data of this type can be computationally compared to single-nucleotide polymorphism (SNP) genetic markers to determine which regions of the mouse genome modulate sedative vs. excitatory effects of ethanol (2.25 mg/kg) in this family.

**Figure 1B:**
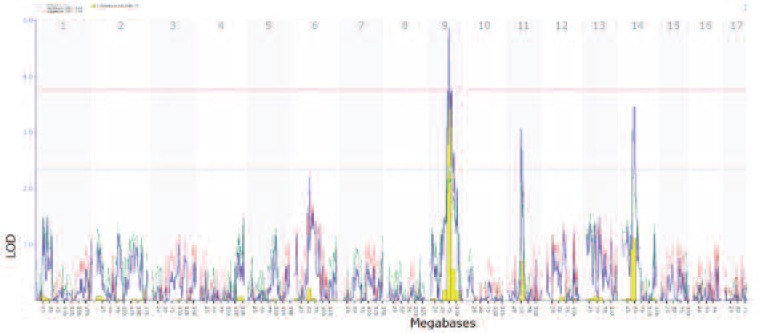
A genetic map of alcohol sedation/activation effects. The horizontal x-axis lists mouse chromosomes, from 1 to the left to chromosome X to the right. The large peak on chromosome 9—a so-called quantitative trait locus (QTL)—is the principal part of the mouse genome that modulates activation levels in females of this BXD family. This sharp peak (high LOD score of 5.0 on the y-axis) can be expanded in GeneNetwork and reveals approximately 160 genes at the peak between 67 and 87 megabases. This set of genes can then be analyzed in GeneWeaver, WebGestalt, COGA, and many other Web resources to evaluate which subsets are most likely to cause differences in response to alcohol, including the suspected alcohol candidate genes, serotonin 1B receptor (5HT1B) and RAB27A. Other features of this genetic map are explained on the Web site.
